# Population size and self-reported characteristics and sexual preferences of men-who-have-sex-with-men (MSM) in Germany based on social network data

**DOI:** 10.1371/journal.pone.0212175

**Published:** 2019-02-14

**Authors:** Stefan Michael Scholz, Oliver Damm, Svenja Elkenkamp, Ulrich Marcus, Wolfgang Greiner, Axel Jeremias Schmidt

**Affiliations:** 1 School of Public Health, Dept. Health Economics and Health Management, Bielefeld University, Bielefeld, Germany; 2 Center for Health Economics Research Hannover (CHERH), Leibniz University Hannover, Hannover, Germany; 3 Robert Koch Institute, Dept. Infectious Diseases Epidemiology, Berlin, Germany; 4 Sigma Research, London School of Hygiene and Tropical Medicine, London, United Kingdom; University of New South Wales, AUSTRALIA

## Abstract

**Background:**

In the absence of detailed information about the population size and behaviour data of men-who-have-sex-with-men (MSM), the estimation of prevalence rates of sexually transmitted infections (STIs) and the design of public health interventions become difficult. The aim of the present study is to estimate the lower boundary of age-specific population sizes and retrieve self-reported information from this population.

**Methods:**

We used publicly accessible data from a large online dating and social network website for MSM in Germany to retrieve data on the age and regional distribution of profiles. The profiles were also stratified by their information on the preferred position during anal intercourse, safer sex, and sexual identity.

**Results:**

A total of 464,873 user profiles correspond to an average 15.2 profiles per 1,000 male inhabitants in Germany, varying between 7.6 and 45.6 across federal states. Although the information on the absolute numbers for different age groups is limited by the search engine, age-specific relative frequencies were found to increase from 12.9 in the age group of 18 to 20 year olds to 24.6 profiles per 1,000 male inhabitants in the 28 to 30 year olds. The data shows age-specific trends for safer sex with an increasing easiness of reporting “never” engaging in safer sex or stating that safer sex “needs discussion” with increasing age. Around one third of profile owners stated to be versatile with respect to the preferred position in anal intercourse. All other options (“only bottom”, “more bottom”, “only top”, “more top”) were preferred equally likely by roughly 10% of profile owners, respectively.

**Conclusions:**

Online social network or dating sites can provide some information about specific populations in the absence of other data sources. The presented results are the first to report age-specific rates of MSM per 1,000 male inhabitants in Germany and may be useful to estimate age-specific prevalence or incidence rates as well as to inform health promotion activities and modelling studies for MSM in Germany.

## Background

Since the beginning of the new millennium and especially since 2010, an increase in diagnosed sexually transmitted infections (STIs) has been observed in many industrialized countries. In this context, men-who-have-sex-with-men (MSM) show higher case numbers for STIs like syphilis or gonorrhoeae than men-who-have-sex-with-women-only (MSW) [1,2]. A major problem for transforming those case numbers into MSM-specific incidence or prevalence rates is the unknown total number of MSM (also known as the "denominator problem" [3]). This makes it also difficult to identify sub-groups at a population level that contribute to the increasing case numbers, and thus would be eligible for tailored health interventions (e.g., health promotion for STI-testing or behaviour change). The impact of such interventions can be assessed in two ways: Firstly, relying on survey data to detect behaviour change over time. Surveys, like *The European Men-Who-Have-Sex-With-Men Internet Survey* (EMIS) [4, 5], often use online platforms to recruit MSM and can cover sexual behaviour associated with the acquisition and transmission of STIs in detail. Secondly, by relying on mathematical modelling, which can simulate the anticipated effect of an intervention including indirect effects. Such models can be useful to link behaviour data collected in surveys to surveillance data of STIs (e.g., [6]). Unfortunately, the lack of data for the calculation of incidence or prevalence rates also makes it difficult to build internally and externally valid models. Model parameters of disease progression or diagnosis and treatment may need to be set to unrealistic values in order to reproduce the case numbers of surveillance data, if the size of the model population is over- or underestimated. Furthermore, models may need to include a stratification of the population into regional, age or sexual-activity groups to allow for the evaluation of interventions designed for any of those sub-groups. These stratifications increase the demand for data and it can be difficult to obtain model parameters of sexual behaviour, like condom usage or the preference of the position in sexual intercourse, for single groups as the number of survey participants in these groups might become relatively small. This may leave modellers to the choice between simpler models or to the extensive use of assumptions weakening the validity of the model.

One possibility to overcome these problems is the use of data from online (dating) platforms, which–as mentioned above–also serve for the recruitment of survey participants [7, 8]. The use of user data of online social networks has become increasingly popular in the social sciences, even with teaching books covering how to analyse major online social networks like Twitter, Facebook or LinkedIn [9]. Topics in the so-called computational social sciences [10] range from the creation of psychological profiles of political extremists [11] to the prediction of the Body Mass Index from profile pictures of users [12] and include even interventional studies altering the emotional content of some Facebook-users’ newsfeed in order to observe their emotional state [13]. In the context of STI research, data from online sites has been used to analyse the risk behaviour of sex workers in the US [14] and in Germany [15]. Online dating apps have also been used to promote health messages for syphilis screening for MSM [16] and to analyse the risk behaviour associated with the use of online dating among MSM [17, 7]. To our knowledge, there is no study so far that makes use of data from MSM dating platforms to provide estimates for the use in the evaluation of health interventions and prevalence and incidence estimations.

The aim of this study, by using data from a large MSM online dating and social network site, is twofold:

1. To estimate the lower boundary of the age-specific population size and regional distribution of MSM in Germany,
2. To provide additional information on general demographics, sexual identity, and self-reported sexual preferences relevant for modelling the impact of interventions among MSM.

## Methods

### Data source

We used data from PlanetRomeo, a large online dating and social network for MSM. Among all the websites used for recruiting participants for the EMIS study, PlanetRomeo contributed 83.3% of all German survey participants [18] and was chosen as it represents the most heavily used website for MSM in Germany. According to the web-traffic analytics of similarweb.com PlanetRomeo ranks 123^rd^ in the list of most used websites in Germany with approximately 8 million visits, with the website www.dbna.de which contributed the second most participants to EMIS only reaching 270,000 visits and ranking 12.829^th^ among the most used websites in Germany. In January 2016, we used the website’s search engine (which at the time was accessible to non-registered users) to count and analyse user profiles from Germany. Via this search function, profiles of registered users can be stratified by certain profile characteristics, and the number of profiles and the corresponding list of all user profiles matching the search strategy are displayed accordingly. Additionally, the number of profiles stratified by federal state was stated on the website.

We searched for every possible combination of the following terms: federal state, self-reported age (between 18 and 75 years), sexual identity (“gay”, “bisexual”, “transgender", or “no entry”), preferred position in anal intercourse (“top only”, “more top”, “versatile”, “more bottom”, “bottom only”, “no” [anal intercourse], or “no entry”), and safer sex (“always”, “needs discussion”, “never”, or “no entry”). A “top” is a person who usually engages in the insertive role in anal intercourse while a “bottom” is a person who prefers to take the receptive role. “Versatile” refers to an individual who may take both parts. PlanetRomeo does not define safer sex and does not give users the possibility to state how they protect themselves and others (e. g. condoms, PrEP). No other terms than the ones reported above were available within the search categories.

The PlanetRomeo search engine poses two problems. First, it principally limits results to 600 hits. In this case, we repeated the search using an additional search term, such as “hair colour”, to reduce the number of hits (for example, if the search term for “18 to 20” year old profiles in “Northrhine-Westfalia”, indicating to be “top” and “always” practising safer sex returns more than 600 hits, the search was split up in different searches additionally including the terms “blonde”, “black”, etc. for hair colour). The corresponding results were combined afterwards and the number of hits for each search was documented in a dataset. The second problem is the overlap of age groups resulting in non-distinct groups. I. e., when searching for 18 to 20 year olds and 20 to 24 year olds, profiles of 20 year olds will be counted twice. This poses a minor problem for the analyses of age-specific trends in behavioural data, but a major problem for the estimation of total age-specific numbers of MSM. However, by standardizing by the general male population of Germany according to data from the German Federal Statistical Office for the year 2015 [19] for each age group, by mirroring this overlap in the denominator, correct age-specific rates of MSM per 1,000 male inhabitants can be reported.

We report the relative frequency of each variable, and display cross tables of two queried characteristics where appropriate. To test for non-random group differences, the results of a chi-squared test will be provided for the latter with the degrees-of-freedom (df) and the corresponding p-value. No technical barriers were breached and no user log-in was necessary to retrieve the data at the time of data collection. Due to a re-design of the website, there is no longer public access to the data. The complete dataset can be found in S1 File.

### Ethics and consent to participate

No individual profiles were accessed for the data retrieval and no individual/personal information has been retrieved as only the number of hits for specific searches was recorded. This is similar to using the number of google hits, which is often used in journalism to underline the importance of the search term or from previous research using only counts of online profiles (e.g., twitter accounts or tweets). We did not gain permission to use the data from the owners/administrators of the website as they provided public access to the data for the general public. No registration or other form of identification was necessary to access the search engine of the website. A screenshot that shows the access of the search engine without being logged in is available upon request. Additionally, at the time of the data gathering, no terms&condition link is provided on the website. This can still be seen on the “classic” design version of the website (https://classic.planetromeo.com/) as well as on previous versions of the website stored in the webarchive (http://web.archive.org/web/20141031080702/https://www.planetromeo.com/).

As we did not access individual profiles, we did not seek consent to participate from the profile/data owners. Using this procedure, it is also impossible to re-identify any profile owner as the collected dataset does not contain information on an individual level. Furthermore, users of PlanetRomeo need to choose whether their profile information will be displayed to the public (i.e., unregistered users) or to remain anonymous to the public when creating a profile on the website.

## Results

### Overall PlanetRomeo users

Overall, as of October 2017, the website stated to have 464,873 registered users with a regional distribution across the federal states of Germany as displayed in Table 1. The highest proportion of profiles among German men was found in Berlin with 45.9 profiles per 1,000 male inhabitants, followed by the other two federal city states of Hamburg and Bremen with 32.8 and 23.4 profiles per 1,000 male inhabitants. All other German federal states showed smaller relative numbers, ranging from 7.6 in Brandenburg to 16.3 per 1,000 male inhabitants in Hesse.

**Table 1:** Number of user profiles in Germany stratified by federal state of residence as stated on the website (as of October 2017) and as retrieved from the search engine (as of January 2016). Due to limitations of the search engine, age groups were overlapping, and thus the total number of profiles is higher than the (correct) total of profiles as stated on the website.

Federal state	Number of profiles (%) from website	Per 1,000 male inhabitants (18–75 years)	Number of profiles (%) from search engine	Concordance
Brandenburg (BB)	7,085 (1.5%)	7.6	7,284 (1.4%	97.3%
Berlin (BER)	60,940 (13.1%)	45.9	72,986 (13.5%)	83.5%
Baden-Wuerttemberg (BW)	51,128 (11.0%)	12.6	59,932 (11.1%)	85.3%
Bavaria (BY)	68,126 (14.7%)	14.2	78,977 (14.6%)	86.3%
Bremen (HB)	5,874 (1.3%)	23.4	7,024 (1.3%)	83.6%
Hesse (HE)	37,411 (8.1%)	16.3	44,353 (8.2%)	84.4%
Hamburg (HH)	21,812 (4.7%)	32.8	26,415 (4.9%)	82.6%
Mecklenburg-Western Pomerania (MV)	6,958 (1.5%)	11.4	7,530 (1.4%)	92.4%
Lower Saxony (NS)	32,818 (7.1%)	11.2	37,083 (6.9%)	88.5%
North Rhine-Westphalia (NW)	100,282 (21.6%)	15.3	117,346 (21.7%)	85.5%
Rhineland-Palatinate (RP)	18,489 (4.0%)	12.3	20,565 (3.8%)	89.9%
Saxony-Anhalt (SA)	8,554 (1.8%)	10.1	9,652 (1.8%)	88.6%
Schleswig-Holstein (SH)	10,901 (2.3%)	10.4	11,773 (2.2%)	92.6%
Saarland (SL)	5,634 (1.2%)	15.1	6,475 (1.2%)	87.0%
Saxony (SX)	19,913 (4.3%)	13.2	23,042 (4.3%)	86.4%
Thuringia (TH)	8,948 (1.9%)	10.9	10,429 (1.9%)	85.8%
TOTAL	464,873 (100%)	15.2	540,866 (100%)	86.0%

Retrieving data via the search engine of the website resulted in a higher number of 540,866 profiles for the age range from 18 to 75 years (as of January 2016). As explained in the methods section, this 1.16-fold overestimation was caused by the overlapping age group definitions in the search engine. The complete distributions of all variables can be found in Tables 2 and 3. About 8% of all profiles (41,102) had no information on the collected variables besides the mandatory information about age and federal state.

**Table 2:** Number of user profiles in Germany stratified by age group as retrieved by the search engine. Due to limitations of the search engine, age groups are overlapping and thus the total number of profiles is higher than the (correct) total of profiles.

	Original results (overlapping age groups)	
Age group	No. profiles (%)	Per 1,000 male inhabitants
18 to 20	17,775 (3.3%)	12.9
20 to 22	21,480 (4.0%)	15.4
22 to 24	27,536 (5.1%)	18.6
24 to 26	36,165 (6.7%)	22.2
26 to 28	39,219 (7.3%)	23.2
28 to 30	40,333 (7.5%)	24.6
30 to 32	36,383 (6.7%)	23.0
32 to 34	33,071 (6.1%)	20.8
34 to 36	35,902 (6.6%)	23.0
36 to 38	33,460 (6.2%)	22.4
38 to 40	31,528 (5.8%)	21.8
40 to 42	27,423 (5.1%)	19.2
42 to 44	25,860 (4.8%)	16.4
44 to 46	29,485 (5.5%)	16.3
46 to 48	27,155 (5.0%)	13.6
48 to 53	41,062 (7.6%)	9.7
53 to 56	15,832 (2.9%)	6.0
56 to 60	11,022 (2.0%)	3.8
60 to 65	6,489 (1.2%)	2.2
65 to 75	3,686 (0.7%)	0.8
TOTAL	540,866 (100%)	13.2

**Table 3:** Characteristics of the user community in Germany including sexual identity, preferred position in anal intercourse and declared commitment to safer sex. Due to limitations of the search engine, age groups are overlapping and thus the total number of profiles is higher than the (correct) total of profiles.

Variable	Optional information	Number of profiles	Percentage
Sexual identity	Gay	279,285	51.6%
	Bisexual	151,969	28.0%
	Transgender	5,012	0.9%
	No entry	104,600	19.3%
	TOTAL	540,866	100.0%
Preferred position [in anal intercourse]	Top only	59,936	11.1%
	More top	43,581	8.1%
	Versatile	177,950	32.9%
	More bottom	58,958	10.9%
	Bottom only	53,889	10.0%
	No [anal intercourse]	14,654	2.7%
	No entry	131,898	24.4%
	TOTAL	540,866	100.0%
Safer sex	Always	322,936	59.7%
	Needs discussion	71,631	13.2
	Never	4,939	0.9
	No entry	141,360	26.1%
	TOTAL	540,866	100.0%

**Fig 1:**
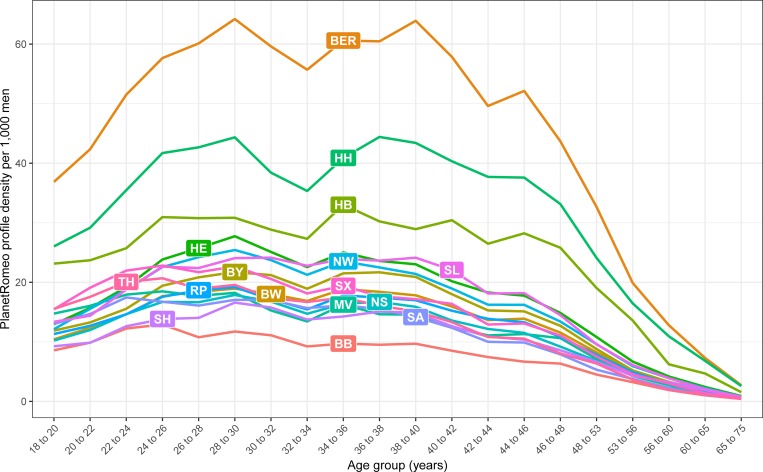
Relative frequency of German accounts over age across the 16 federal states. Relative frequency standardised by the respective male population. Due to limitations of the website’s search engine, age groups are overlapping. The specific values can be found in S1 Table.

With regard to the age groups, the relative number of profiles per 1,000 male inhabitants started at a level of 12.9 profiles for 18 to 20 year-old men in Germany. The relative density of profiles increased with increasing age to the maximum in the age group of 28 to 30 year-olds (24.6). Beyond 40 years of age, the profile density dropped below 19.2 and decreased to 0.8 profiles per each 65 to 75 year-old men. Fig 1 shows the distribution of the profiles over the different age groups stratified by federal states. The highest profile density among men was found among 28 to 30 year-olds (64.2) and 38 to 40 year-olds (63.9) in Berlin.

### Sexual identity and sexual behaviour

For all optional characteristics (sexual identity, “position [in anal intercourse]”, and safer sex), the proportion of profiles with missing information was 19.3%, 24.4% and 26.1%, respectively, across all age groups. For all variables, missing values decreased with increasing age. With regard to sexual identity, this proportion decreased significantly from 30.4% of profiles stating no information about their sexual identity in the age group 18–20 years (40.2% "gay", 28.6% "bisexual", and 0.8%"transgender") to 8.7% in the oldest age group (df = 1, p-value < 0.001). While the proportions of profiles stating to be "bisexual" or "transgender" remained relatively stable at around 25%, the share of profiles indicating the owner to identify as “gay" increased from 40.2% to 64.8% (df = 1, p-value < 0.001) from the youngest to the oldest age group (26.1% "bisexual" and 0.3% "transgender"). Transgender rates varied only little (between 0.10% and 0.13%) between 20 and 34 year-olds (df = 6; p-value = 0.001), but decreased with age for older age groups. There seemed to be a tendency for profiles of the federal city states Berlin and Hamburg to have a significantly higher probability of indicating to be "gay" (63.1% and 57.4%, respectively; df = 2, p-value < 0.001), while transgender and no information about the sexual orientation showed the same relative frequency as in the other federal states.

The preferred position in anal intercourse was distributed relatively symmetrical over the top-bottom-scale. A majority of profiles indicated a preference for both roles (“versatile” 32.9%), while "more top" (8.1%) and "more bottom" (10.9%) and "top only" (11.1%) and "bottom only" (10.0%) showed rather similar proportions. Overall, 2.7% of all profiles stated to have no interest in anal intercourse. The "no anal intercourse" self-statements are highly age-dependent and their relative frequency increased from 0.7% in the age group 18–20 to 11.3% in the age group 65–75 (df = 1; p-value < 0.001). Besides the decrease of the profiles with "no information" on their position, a clear age-dependency was found in the "top only" category increasing from 5.7% in the youngest to 13.3% in the age group 53–56 (df = 1; p-value < 0.001). All other categories showed no apparent age-dependency. In addition, no association was found between the preferred position in anal intercourse and federal state or sexual identity, besides profiles indicating a “transsexual” identity preferring “more bottom” or “bottom only” positions.

**Fig 2:**
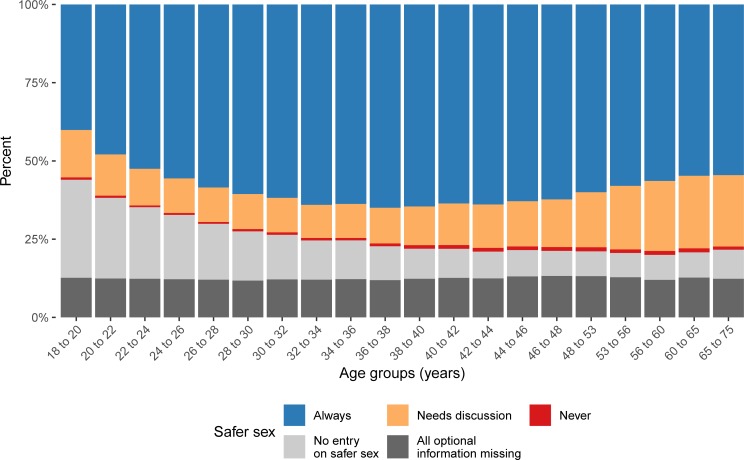
Safer sex across age. Relative frequency of safer sex behaviour over all age groups. Due to limitations of the website’s search engine, age groups are overlapping. The specific values can be found in S2 Table.

**Fig 3:**
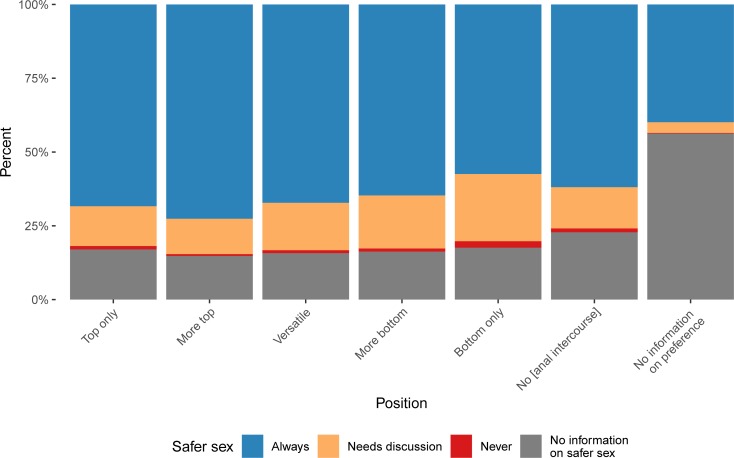
Safer sex and preferred position. Relative frequency of safer sex intentions according to preferred position in anal intercourse. The specific values can be found in S3 Table.

As can be seen in Fig 2, the age-dependency found for sexual identity and for the preferred position in anal intercourse was also apparent in the profile information about safer sex. On first sight, the decrease of missing information in the profiles with increasing age of the owner is also apparent. When stratifying the “no entry” profiles, the number of profiles containing no information about the preferred position in anal intercourse and no information about safer sex remained at around 12% across all age groups (“All optional information missing”). The number of persons without information on safer sex, but stating their preferred position in anal intercourse, decreased with increasing age, represented by the “No entry on safer sex” category in Fig 3. The proportion of profiles, stating to never practice safer sex, increased from 0.5% in the age group 22 to 24 years to 1.3% in the age group 60 to 65 years (df = 1; p-value < 0.001). The statement "needs discussion" showed a very similar pattern with an increase from 11.7% to 23.2% from the age group 22 to 24 to the age group 60 to 65 years (df = 1; p-value < 0.001). However, in the youngest age groups under 22 years, the proportions of both statements (“never” and “needs discussion”) were significantly higher than for the 22 to 24 year olds (df = 1; p-value < 0.001) and significantly lower than for the 65 to 75 year olds (df = 1; p-value < 0.001).

In the combination of the statements on the preferred position in anal intercourse and safer sex, it becomes apparent that the relative frequency of profiles stating to always practice safer sex decreased with a more receptive role in anal intercourse (see Fig 3). In the "bottom only" group 2.2% stated to never practice safer sex and 22.8% stated “needs discussion”, leaving 57.4% stating that they always practice safer sex. “Top only” and “more top” showed the highest share of profiles stating to always practice safer sex (68.4 and 72.6%, respectively). No clear regional trends regarding safer sex were found.

## Discussion

Using data from the largest German online social network and dating site for MSM, a lower bound of at least 464,873 MSM between the age of 18 and 75 could be found, corresponding to 1.52% of the respective (18–75) male adult population. The highest proportion was found in the age group of 28 to 30 year-olds, and the number and the proportion of profiles decreased after the age of 46. The age distributions showed slight “bumps” before the age of 30 and 40 years, which may be due to internet users being deceptive about their age [20]. Our results are comparable to the results of Marcus et al. (2009)[3], which give estimates of the regional distribution of MSM between 20 and 59 years in 2006, ranging between 574.750 and 786.500. A detailed comparison to this study by federal states can be found in S4 Table, but, besides the double counting of some ages, our estimates exceed the number of MSM in the lower scenario only in five federal states with the maximal deviance of 21.1% found for Bavaria (75.809 vs. 62.600).

With regard to the optional information, more than half of the profiles owners indicated to be "gay" and 28.1% to be "bisexual". In this context, it should be stated that “transgender” does not reflect sexual orientation such as “gay” or “bisexual” but rather a gender identity. The presence of “transgender” as the only other option given by the website might have pushed transgender persons to mis-identify their sexual orientation as “transgender”. A higher number and proportion of profiles were found in metropolitan areas, and more users identified as “gay” in those areas. This may be due to the migration of MSM into larger cities. Most profiles (about one third) indicated equally preferring an insertive or receptive position in anal intercourse, respectively, with around ten percent preferring either a “rather active” or a “rather passive” role, or an “only active” or an “only passive” role, respectively. Around 60% of profiles indicated to practice safer sex, and only less than one percent stated to never practice safer sex. With age, the share of persons practicing safer sex, or only “by discussion”, increases.

There are two issues that need to be considered interpreting the analyses of the behavioural aspects. Firstly, there is a relatively high amount of profiles with no information (“no entry”) which may not be missing at random, thus being a potential source of bias. Secondly, certain terminologies (e.g., “safer sex”) might be subject to a variety of definitions by different users, making a common interpretation difficult. The strong age-dependency could indicate two different phenomena. The higher share of "no information" profiles in the younger age groups may be due to a higher awareness for data privacy issues, leading to users who are unwilling to reveal personal information that are perceived to be of high sensitivity. Another reason may be indifference about certain variables at a young age that might become clearer with increasing age (e.g., sexual identity). The relatively high relative frequency of persons in the age group from 18 to 20 indicating to perform safer sex "never" or only according to a negotiated agreement ("needs discussion") might be due to younger MSM not knowing the community’s common understanding of safer sex or having a stronger preference for monogamous relationships with seroconcordant partners. The increase of users stating that they practice safer sex "never" or based on a negotiated agreement ("needs discussion") in the older age groups may be attributed to a higher share of HIV-positive men in these age groups who engage in serosorting, meaning that a person prefers to choose sexual partners based on HIV status. However, motivational reasons for engaging in intentional unprotected anal intercourse (bareback sex) are diverse, and barebacking is not limited to HIV-positive MSM. An interview study conducted in five European and North American cities found that for some MSM semen exchange includes a symbolic role leading to a higher level of connectedness, completion, or naturalness [21]. Apart from such interpersonal processes, medical advances leading to a reduced risk of infection from HIV positive partners using combination antiretroviral therapy (cART) and therefore having undetectable viral load [22, 23, 24, 25] may contribute to condomless anal sex. Other factors may contribute to barebacking [26] and/or negotiated risk [27] and cover the Internet as a facilitating factor of negotiated risk [28], lack of community activism, lower perception of safer sex norms, and intrapersonal factors like sociodemographic characteristics (e.g., low education) [29] and personality traits (e.g., low level of perceived responsibility, desire for sexual pleasure, or sexual sensation seeking) [30]. The difference between “tops” and “bottoms” with regards to their safer sex behavior may not necessarily indicate that bottoms are more open to condomless anal intercourse; it may just indicate the different capacity for decision-making. Since the top and not the bottom has to use the condom, the bottom necessarily has to discuss condom use with the top if he wants to make sure that condoms are used, while the top can just use a condom without discussing it. However, also tops might need to discuss condom use or safer sex in general.

The presented results have several limitations. It remains unclear what percentage of the MSM population owns a profile on the studied website and if there are differences in the use of online services between certain groups (e.g., age). The latest survey among MSM in Germany found that 79% of all survey participants used chat, dating, or contact online services monthly or even several times a week. 9% of participants over 44 years of age did not use any online service while 5% to 6% in the younger age groups stated to never use online services [31]. It may also be possible that a single user might have several profiles for different regions, e.g. one for his work place and one for the location of residence or for different purposes (long-term relationship vs. casual dating). In summary, it is not possible to determine the proportion of gay and bisexual men that use PlanetRomeo and to incorporate this in the calculation of the overall number of MSM in Germany. Thus it is only possible to interpret our estimations as lower boundaries. Furthermore, the data does not allow to identify profiles that are connected to a certain region only for a short period of time (e.g., vacation, business trip, etc.). These limitations would allow both for under- and overestimation of the lower boundary of the number of MSM in Germany and may also be a biasing factor when analysing the regional distribution of MSM. In this context, the low relative, standardised frequency in the state Brandenburg may be the result of residents of this state to choose the more attractive but close-by state of Berlin instead of their actual place of residence. In general, federal city states have a higher density of profiles. Furthermore, as the data represents online, self-reported data, the numbers may significantly differ from the actual behaviour in reality and need to be interpreted with caution.

## Conclusions

In the absence of other data sources, social media websites may to be one way to retrieve information about lower limits of population sizes and behaviour data of specific populations. The present study provided numbers on the total population size and age-specific rates of MSM in Germany as well as sub-group-specific information on stated behaviour. This data can be used for tailoring health promotion and to inform modelling studies, which otherwise might need to strongly rely on assumptions on the population size and behavioural parameters.

## Supporting information

S1 File. Dataset Dataset containing the collected data from PlanetRomeo. (CSV)

S1 Table Relative frequency of German accounts. Relative frequency of German accounts over age across the 16 federal states, standardised by the respective male population. Due to limitations of the website’s search engine, age groups are overlapping.
(DOCX)

S2 Table Safer sex across age. Relative frequency of safer sex behaviour over all age groups. Due to limitations of the website’s search engine, age groups are overlapping.
(DOCX)

S3 Table Safer sex across preferred position. Relative frequency of safer sex intentions according to preferred position in anal intercourse.
(DOCX)

S4 Table Comparison of population size. Comparison between the estimates of the population sizes of MSM between 20 and 59 years in the 16 federal states of Germany between the present study and Marcus et al. (2009).
(DOCX)

## Abbreviations

MSM: men having sex with men
STIs: sexually transmitted infections
MSW: men having sex with women only
EMIS: The European Men-Who-Have-Sex-With-Men Internet Survey
PrEP: pre-exposure prophylaxis
BB: Brandenburg
BER: Berlin
BW: Baden-Wuerttemberg
BY: Bavaria
HB: Bremen
HE: Hesse
HH: Hamburg
MV: Mecklenburg-Western Pomerania
NS: Lower Saxony
NW: North Rhine-Westphalia
RP: Rhineland-Palatinate
SA: Saxony-Anhalt
SH: Schleswig-Holstein
SL: Saarland
SX: Saxony
TH: Thuringia
HIV: human immunodeficiency virus
