# Supplementary material for: Population size and self-reported characteristics and sexual preferences of men-who-have-sex-with-men (MSM) in Germany based on social network data
Source: PLoS One. 2019 Feb 14;14(2):e0212175. doi: 10.1371/journal.pone.0212175 (PMC6375596; doi:10.1371/journal.pone.0212175)
Supplement: S1 Table — Relative frequency of German accounts over age across the 16 federal states, standardised by the respective male population. Due to limitations of the website’s search engine, age groups are overlapping. (DOCX) [file pone.0212175.s002.docx]

S1 Table: Relative frequency of German accounts over age across the 16 federal states, standardised by the respective male population. Due to limitations of the website’s search engine, age groups are overlapping.

| **AREA** | **BB** | **BER** | **BW** | **BY** | **HB** | **HE** | **HH** | **MV** | **NS** | **NW** | **RP** | **SA** | **SH** | **SL** | **SX** | **TH** |
| --- | --- | --- | --- | --- | --- | --- | --- | --- | --- | --- | --- | --- | --- | --- | --- | --- |
| **18 to 20** | 8.58 | 36.87 | 10.43 | 11.89 | 23.14 | 12.96 | 26.04 | 14.75 | 10.23 | 12.13 | 11.32 | 13.04 | 9.26 | 13.37 | 15.47 | 15.53 |
| **20 to 22** | 9.88 | 42.35 | 12.36 | 13.27 | 23.71 | 15.71 | 29.17 | 16.06 | 11.99 | 15.56 | 12.70 | 14.68 | 9.85 | 14.40 | 19.10 | 17.56 |
| **22 to 24** | 12.25 | 51.52 | 14.63 | 15.56 | 25.74 | 19.20 | 35.48 | 17.93 | 14.66 | 18.75 | 14.64 | 17.46 | 12.63 | 18.78 | 21.96 | 20.08 |
| **24 to 26** | 12.92 | 57.66 | 17.68 | 19.43 | 30.95 | 23.84 | 41.71 | 18.49 | 16.71 | 22.57 | 17.57 | 16.74 | 13.85 | 22.64 | 22.82 | 20.68 |
| **26 to 28** | 10.78 | 60.12 | 18.41 | 20.84 | 30.78 | 25.74 | 42.67 | 17.73 | 16.66 | 24.22 | 18.67 | 16.10 | 14.03 | 22.40 | 21.71 | 18.91 |
| **28 to 30** | 11.74 | 64.21 | 18.94 | 21.73 | 30.84 | 27.74 | 44.35 | 18.24 | 17.88 | 25.43 | 19.17 | 17.06 | 16.60 | 24.06 | 22.67 | 19.57 |
| **30 to 32** | 11.11 | 59.62 | 18.04 | 21.21 | 28.85 | 25.10 | 38.41 | 15.28 | 16.77 | 23.74 | 17.15 | 17.03 | 15.77 | 24.15 | 20.57 | 17.38 |
| **32 to 34** | 9.24 | 55.71 | 16.88 | 18.93 | 27.31 | 22.56 | 35.34 | 13.43 | 14.72 | 21.26 | 15.45 | 15.67 | 13.76 | 22.78 | 18.13 | 16.70 |
| **34 to 36** | 9.72 | 60.61 | 18.88 | 21.51 | 33.03 | 25.02 | 40.89 | 16.27 | 16.41 | 23.53 | 17.80 | 16.15 | 14.26 | 23.93 | 19.42 | 17.45 |
| **36 to 38** | 9.52 | 60.47 | 18.41 | 21.68 | 30.23 | 23.59 | 44.42 | 14.64 | 16.71 | 22.50 | 17.44 | 15.52 | 15.09 | 23.65 | 17.65 | 16.06 |
| **38 to 40** | 9.69 | 63.92 | 17.81 | 20.90 | 28.93 | 23.03 | 43.41 | 14.57 | 15.89 | 21.41 | 16.99 | 14.17 | 14.89 | 24.13 | 17.17 | 15.13 |
| **40 to 42** | 8.52 | 57.87 | 15.94 | 18.01 | 30.44 | 20.19 | 40.33 | 12.45 | 13.59 | 18.95 | 15.24 | 12.37 | 12.63 | 22.08 | 16.38 | 13.32 |
| **42 to 44** | 7.45 | 49.62 | 13.66 | 15.28 | 26.48 | 18.29 | 37.71 | 11.09 | 12.17 | 16.24 | 13.87 | 10.01 | 10.88 | 18.13 | 12.91 | 10.83 |
| **44 to 46** | 6.68 | 52.14 | 13.90 | 15.12 | 28.23 | 17.77 | 37.59 | 11.31 | 11.52 | 16.22 | 13.30 | 9.89 | 10.38 | 18.18 | 13.10 | 10.54 |
| **46 to 48** | 6.35 | 43.69 | 11.56 | 12.70 | 25.81 | 14.86 | 33.15 | 10.63 | 9.20 | 13.42 | 10.84 | 7.91 | 8.58 | 14.48 | 11.07 | 8.05 |
| **48 to 53** | 4.51 | 32.66 | 8.23 | 8.79 | 19.05 | 10.82 | 24.11 | 7.13 | 6.81 | 9.62 | 7.83 | 5.33 | 6.66 | 9.59 | 7.53 | 6.37 |
| **53 to 56** | 3.24 | 19.89 | 5.15 | 5.21 | 13.60 | 6.69 | 16.47 | 4.26 | 4.43 | 5.93 | 4.91 | 3.77 | 4.30 | 6.11 | 4.57 | 3.66 |
| **56 to 60** | 1.91 | 12.79 | 3.28 | 3.27 | 6.23 | 4.19 | 10.94 | 2.74 | 3.04 | 3.90 | 2.97 | 2.29 | 3.22 | 3.89 | 2.99 | 1.96 |
| **60 to 65** | 1.03 | 7.30 | 1.93 | 1.99 | 4.69 | 2.43 | 6.82 | 1.24 | 1.66 | 2.21 | 1.62 | 1.09 | 1.93 | 2.07 | 1.39 | 1.15 |
| **65 to 75** | 0.42 | 2.72 | 0.81 | 0.75 | 1.55 | 0.90 | 2.58 | 0.61 | 0.67 | 0.81 | 0.57 | 0.47 | 0.73 | 0.77 | 0.57 | 0.41 |
