# Supplementary material for: Population size and self-reported characteristics and sexual preferences of men-who-have-sex-with-men (MSM) in Germany based on social network data
Source: PLoS One. 2019 Feb 14;14(2):e0212175. doi: 10.1371/journal.pone.0212175 (PMC6375596; doi:10.1371/journal.pone.0212175)
Supplement: S2 Table — Relative frequency of safer sex behaviour over all age groups. Due to limitations of the website’s search engine, age groups are overlapping. (DOCX) [file pone.0212175.s003.docx]

S2 Table: Relative frequency of safer sex behaviour over all age groups. Due to limitations of the website’s search engine, age groups are overlapping.

| **AGE** | **Always** | **Needs discussion** | **Never** | **No entry  on safer sex** | **All optional  information missing** |
| --- | --- | --- | --- | --- | --- |
| **18 to 20** | 0.401 | 0.151 | 0.008 | 0.313 | 0.127 |
| **20 to 22** | 0.479 | 0.132 | 0.007 | 0.258 | 0.124 |
| **22 to 24** | 0.525 | 0.117 | 0.005 | 0.229 | 0.124 |
| **24 to 26** | 0.556 | 0.111 | 0.006 | 0.205 | 0.122 |
| **26 to 28** | 0.585 | 0.110 | 0.006 | 0.179 | 0.121 |
| **28 to 30** | 0.606 | 0.112 | 0.007 | 0.157 | 0.118 |
| **30 to 32** | 0.618 | 0.110 | 0.008 | 0.143 | 0.121 |
| **32 to 34** | 0.640 | 0.106 | 0.008 | 0.126 | 0.121 |
| **34 to 36** | 0.637 | 0.109 | 0.008 | 0.124 | 0.122 |
| **36 to 38** | 0.650 | 0.114 | 0.009 | 0.108 | 0.119 |
| **38 to 40** | 0.645 | 0.124 | 0.011 | 0.096 | 0.124 |
| **40 to 42** | 0.636 | 0.133 | 0.012 | 0.093 | 0.126 |
| **42 to 44** | 0.639 | 0.139 | 0.012 | 0.086 | 0.125 |
| **44 to 46** | 0.629 | 0.144 | 0.012 | 0.084 | 0.131 |
| **46 to 48** | 0.623 | 0.153 | 0.012 | 0.080 | 0.132 |
| **48 to 53** | 0.600 | 0.176 | 0.013 | 0.079 | 0.132 |
| **53 to 56** | 0.580 | 0.203 | 0.011 | 0.077 | 0.128 |
| **56 to 60** | 0.564 | 0.224 | 0.012 | 0.080 | 0.120 |
| **60 to 65** | 0.547 | 0.232 | 0.013 | 0.081 | 0.127 |
| **65 to 75** | 0.545 | 0.228 | 0.010 | 0.093 | 0.123 |
