# Supplementary material for: Population size and self-reported characteristics and sexual preferences of men-who-have-sex-with-men (MSM) in Germany based on social network data
Source: PLoS One. 2019 Feb 14;14(2):e0212175. doi: 10.1371/journal.pone.0212175 (PMC6375596; doi:10.1371/journal.pone.0212175)
Supplement: S3 Table — Relative frequency of safer sex intentions according to preferred position in anal intercourse. (DOCX) [file pone.0212175.s004.docx]

S3 Table: Relative frequency of safer sex intentions according to preferred position in anal intercourse.

| **Preferred position** | **Always** | **Needs discussion** | **Never** | **No information on safer sex** |
| --- | --- | --- | --- | --- |
| **Top only** | 0.684 | 0.135 | 0.011 | 0.170 |
| **More top** | 0.726 | 0.120 | 0.006 | 0.148 |
| **Versatile** | 0.672 | 0.161 | 0.010 | 0.158 |
| **More bottom** | 0.647 | 0.180 | 0.011 | 0.163 |
| **Bottom only** | 0.574 | 0.228 | 0.022 | 0.176 |
| **No [anal intercourse]** | 0.619 | 0.140 | 0.013 | 0.228 |
| **No information on preference** | 0.399 | 0.036 | 0.002 | 0.563 |
