# Supplementary material for: Population size and self-reported characteristics and sexual preferences of men-who-have-sex-with-men (MSM) in Germany based on social network data
Source: PLoS One. 2019 Feb 14;14(2):e0212175. doi: 10.1371/journal.pone.0212175 (PMC6375596; doi:10.1371/journal.pone.0212175)
Supplement: S4 Table — Comparison between the estimates of the population sizes of MSM between 20 and 59 years in the 16 federal states of Germany between the present study and Marcus et al. (2009). (DOCX) [file pone.0212175.s005.docx]

S4 Table: Comparison between the estimates of the population sizes of MSM between 20 and 59 years in the 16 federal states of Germany between the present study and Marcus et al. (2009).

| **Federal state** | **Present study** | **Marcus et al. (2009)** | | | |  |
| --- | --- | --- | --- | --- | --- | --- |
|  |  | **lower** | **Concordance** | **upper** | **Concordance** | |
| Brandenburg | 6.971 | 6.900 | 101,0% | 10.000 | 69,7% | |
| Berlin | 70.783 | 77.000 | 91,9% | 105.000 | 67,4% | |
| Baden-Wuerttemberg | 57.391 | 62.200 | 92,3% | 85.000 | 67,5% | |
| Bavaria | 75.809 | 62.600 | 121,1% | 85.400 | 88,8% | |
| Bremen | 6.703 | 6.450 | 103,9% | 8.800 | 76,2% | |
| Hesse | 42.663 | 47.500 | 89,8% | 65.000 | 65,6% | |
| Hamburg | 25.474 | 25.500 | 99,9% | 34.500 | 73,8% | |
| Mecklenburg-Western Pomerania | 7.191 | 8.000 | 89,9% | 11.000 | 65,4% | |
| Lower Saxony | 35.312 | 49.500 | 71,3% | 67.700 | 52,2% | |
| Northrhine-Westfalia | 112.704 | 148.000 | 76,2% | 202.500 | 55,7% | |
| Rhineland-Palatinate | 19.630 | 17.200 | 114,1% | 23.500 | 83,5% | |
| Saxony-Anhalt | 9.233 | 9.800 | 94,2% | 13.400 | 68,9% | |
| Schleswig-Holstein | 11.187 | 13.500 | 82,9% | 18.400 | 60,8% | |
| Saarland | 6.210 | 6.700 | 92,7% | 9.100 | 68,2% | |
| Saxony-Anhalt | 22.173 | 25.000 | 88,7% | 35.000 | 63,4% | |
| Thuringia | 9.971 | 8.900 | 112,0% | 12.200 | 81,7% | |
| **TOTAL** | **519.405** | **574.750** | **90,4%** | **786.500** | **66,0%** | |
